# Adaptation of the QoL-AGHDA scale for adults with growth hormone deficiency in four Slavic languages

**DOI:** 10.1186/1477-7525-9-60

**Published:** 2011-08-02

**Authors:** Stephen P McKenna, Jeanette Wilburn, James Twiss, Sigrid R Crawford, Václav Hána, Malgorzata Karbownik-Lewinska, Vera Popovic, Mikulas Pura, Maria Koltowska-Häggström

**Affiliations:** 1 Galen Research Ltd, Manchester, UK; 2 Third Department of Internal Medicine, Charles University, Prague, Czech Republic; 3 Department of Oncological Endocrinology, Medical University of Lodz, Poland; 4 Institute of Endocrinology, University Clinical Centre, Belgrade, Serbia; 5 Department of Endocrinology, National Institute of Endocrinology & Diabetology, Lubochna, Slovakia; 6 Pfizer Endocrine Care, Sollentuna, Sweden

**Keywords:** Adaptation, Validation, QoL-AGHDA, Czech Republic, Poland, Serbia, Slovakia

## Abstract

**Purpose:**

The Quality of Life in Adult Growth Hormone Deficiency Assessment (QoL-AGHDA) is a disease-specific quality of life measure specific to individuals who are growth hormone deficient. The present study describes the adaptation of the QoL-AGHDA for use in the following four Slavic languages; Czech, Polish, Serbian and Slovakian.

**Methods:**

The study involved three stages in each language; translation, cognitive debriefing and validation. The validation stage assessed internal consistency (Cronbach's alpha), reproducibility (test-retest reliability using Spearman's rank correlations), convergent and divergent validity (Correlations with the NHP) and known group validity.

**Results:**

The QoL-AGHDA was successfully translated into the target languages with minimal problems. Cognitive debriefing interviewees (n = 15-18) found the measures easy to complete and identified few problems with the content. Internal consistency (Czech Republic = 0.91, Poland = 0.91, Serbia = 0.91 and Slovakia = 0.89) and reproducibility (Czech Republic = 0.91, Poland = 0.91, Serbia = 0.88 and Slovakia = 0.93) were good in all adaptations. Convergent and divergent validity and known group validity data were not available for Slovakia. The QoL-AGHDA correlated as expected with the NHP scales most relevant to GHD. The QoL-AGHDA was able to distinguish between participants based on a range of variables.

**Conclusions:**

The QoL-AGHDA was successfully adapted for use in the Czech Republic, Poland, Serbia and Slovakia. Further validation of the Slovakian version would be beneficial. The addition of these new lanaguage versions will prove valuable to multinational clinical trials and to clinical practice in the respective countries.

## Background

Adult Growth Hormone Deficiency (GHD) is a medical condition in which the body does not produce enough growth hormone. The prevalence of GHD in adults has been estimated to be as high as 3 in 10,000 in the UK [1]. The condition is associated with abnormalities in body composition [2-4], functional impairment [5] and a number of cardiovascular risk factors [6-8]. The disease has been reported to have a major impact on quality of life (QoL) resulting from increased levels of fatigue [9], social isolation [10], anxiety [11] and impaired memory [12].

In order to measure the impact of replacement GH on patients the Quality of Life Assessment of Growth Hormone Deficiency in Adults (QoL-AGHDA) was developed [13]. The scale consists of 25 statements answered 'Yes' or 'No'. Scores range from 0 to 25 with a high score indicating poor QoL. The content of the QoL-AGHDA was generated directly from patient interviews and new groups of patients were involved in each subsequent stage of the development. The measure adopted the needs-based model of QoL described by Hunt and McKenna in 1992 [14,15]. According to this model life gains its quality from the ability and capacity of the individual to meet his or her needs. QoL is high when an individual is able to meet his or her needs.

The QoL-AGHDA has been widely used in clinical practice and research studies [16-18]. It is also used in KIMS (Pfizer International Metabolic Database), an international research database, that monitors the long-term treatment outcomes and safety of growth hormone replacement therapy [19]. In the UK, the National Institute for Health and Clinical Excellence has recommended that recombinant human growth hormone (somatropin) treatment is given to an adult with GH deficiency only if he or she meets three criteria; that the individual has severe GH deficiency, that he or she is already receiving full replacement with other deficient pituitary hormones as required and that he or she has a perceived impairment of QoL as demonstrated by a score of at least 11 on the QoL-AGHDA [1]. Furthermore, an adult who has been started on GH treatment has to be re-assessed for QoL status nine months after the initiation of therapy. GH treatment is discontinued if the individual has an improvement of fewer than seven points in QoL-AGHDA score. This is the first time that a QoL measure has been used to determine whether or not treatment should be given.

The QoL-AGHDA was originally developed for use in five languages; UK English, Swedish, Italian, German and Spanish [20]. Since then it has also been adapted for use in the United States, Belgium, the Netherlands, Brazil and Denmark. The present paper reports on the development of new language versions of the QoL-AGHDA for use in four Slavic languages. These new language versions will increase the value of the measure to international clinical trials and research studies. In addition, the new language versions could also improve patient management in the individual countries and assist in selection of treatment for patients, as occurs in the UK [1].

The adaptation of any questionnaire into a new language presents researchers with several linguistic and conceptual challenges. Language contains many nuances and phrases that, although well understood in the language in which the instrument was developed, are not always clear to non-native speakers. Consequently, it is inappropriate to produce a new language version of a questionnaire by simply translating the content (literal translation). In order to overcome these adaptation challenges each language version of the QoL-AGHDA is adapted according to a standardised adaptation procedure that uses the dual panel methodology [21].

This approach involves conducting two translation panels; a bilingual panel (to provide the initial translation into the target language) and a lay panel (where items are assessed for comprehension and 'naturalness' of language). The objective in adapting questionnaires is to ensure that items are understood in the same way in different countries. The wording of items is crucial to a respondent's interpretation of the question and to their response. The dual panel methodology emphasises the importance of achieving conceptual equivalence of translated items. It is not always possible to find a 'natural' translation for an item in a new language or, where it is possible, the natural translation sometimes does not mean the same as in the original language. In such circumstances it is necessary to find a phrase that describes an equivalent concept. Linguistic equivalence is of secondary importance in the dual panel methodology. In addition, it is stressed that new items should be expressed in common (everyday) language that will appeal to future respondents. The dual panel methodology has been shown to produce translations that are more acceptable to patients than forward-backward translations [22]. This method has also been successfully applied in the adaptation of other questionnaires into Slavic languages [23].

The present study describes the adaptation of the QoL-AGHDA for use in the following four Slavic languages; Czech, Polish, Serbian and Slovakian.

## Methodology

Three main stages of adaptation were conducted in each country; translation of the questionnaire, cognitive debriefing interviews to establish face and content validity in the new cultures and formal validation by means of a postal survey.

### Participants

Participants in the translation panels were not growth hormone deficient as their role was to ensure the appropriate wording of items in the new language rather than to generate new items for the questionnaire.

Participants in the cognitive debriefing interviews and postal validation surveys were patients diagnosed with growth hormone deficiency recruited at the participating centres:

• Department of Internal Medicine, Charles University, Prague, Czech Republic.

• Department of Oncological Endocrinology, Medical University of Lodz, Poland.

• Institute of Endocrinology, University Clinical Centre, Belgrade, Serbia.

• Department of Endocrinology, National Institute of Endocrinology & Diabetology, Lubochna, Slovakia.

### Translation

The translation method consisted of conducting two panels; a bilingual panel (to provide the initial translation into the target language) and a lay panel (where items are assessed for comprehension and 'naturalness' of language). Each panel consisted of five or six participants who worked as a team and both panels were chaired by the same group leader. His/her role was to encourage the panel members to reach consensus on the appropriate translations for the instructions, items and response options. The leader was also required to ensure that no panel member became too dominant by encouraging each member to voice their opinions. The bilingual panels were also attended by one of the original instrument developers whose role was to explain the precise conceptual meaning of the items to panel members.

The bilingual panels work predominantly in the target language. Items are presented to the groups one-by-one and their meaning explained. Alternative translations suggested by individual group members are considered by the whole group. Each item is discussed until agreement is reached. Where consensus cannot be reached alternative versions of the item are taken forward for consideration by the lay panel.

The lay panels work only in the target language. Individuals are selected for this panel if they have an average or lower than average educational level. The purpose of this second panel is to ensure that the final wording of items is appropriate for the average future respondent. Participants are presented with the translation(s) made by the bilingual panel and asked to comment on it/them in terms of comprehension and acceptability. In particular, they are asked to decide whether phrasing and language are acceptable or whether these should be changed to make the items more 'natural' while maintaining their original meaning. Where necessary they are also asked to choose between alternative translations that the bilingual panel has produced.

### Cognitive debriefing interviews

The purpose of these interviews is to test the applicability, comprehension, relevance and comprehensiveness of the new scales with relevant patients. In the interviews (which are face-face and semi-structured) respondents are asked to complete the questionnaire in the presence of an interviewer who notes any obvious difficulties or hesitation over specific items. Interviewees are then asked to comment on the questionnaire items, instructions and response format. Respondents are also asked whether any aspects of their experience of GH deficiency have been omitted.

### Postal validation survey

Data needed to establish the psychometric properties of the new language versions were collected by means of postal surveys conducted with growth hormone deficient patients in the Czech Republic, Poland and Slovakia. In Serbia patients completed the measures at the participating clinical centre. The specific design of this survey varied from country to country due to the limited availability of validated comparator instruments and local circumstances.

The QoL-AGHDA was administered on two occasions, with two weeks between administrations. The measure has 25 items scored 1 (if affirmed) or 0 if not. Consequently scores can range from 0 to 25 with high scores indicating worse QoL. Participants were also asked to complete the Nottingham Health Profile (NHP) [24] (where available) and a 'KIMS Patient Life Situation Form' (KIMS PLSF) [18] on the first occasion. The latter questionnaire includes demographic questions and ratings of perceived health status and disease severity.

### Statistical analyses

Non-parametric statistical tests were used throughout the analyses due to the ordinal nature of the measures employed. All statistical tests are two-tailed with a *p *value of .05 indicating statistical significance.

The distributional properties of the QoL-AGHDA were explored through descriptive statistics (median and inter quartile range (IQR)), and floor and ceiling effects (percentage of patients scoring the minimum and maximum possible scores, respectively).

Internal consistency is assessed using Cronbach's alpha coefficients. Alpha measures the extent to which the items in a scale are inter-related. A low alpha (below 0.7) indicates insufficient relations between the items [25]. In addition, each item is correlated with the total score (corrected-item total coefficients (CITCs)). If this correlation is low (below 0.2) it can indicate that the item is not contributing adequately to the overall scale. If the correlation is high (above 0.8) it suggests that the item is redundant, adding little extra information to the scale.

The test-retest reliability of a measure is an estimate of its reproducibility over time when no change in condition has taken place. It is assessed here by calculating Spearman rank correlation coefficients on responses to the QoL-AGHDA collected on the two different occasions. A high correlation indicates that the instrument produces low levels of random measurement error. A minimum value of 0.85 is required [26].

QoL-AGHDA scores are compared for males and females and for respondents who are above or below the median age. Mann-Whitney U-tests are employed to test for differences.

Convergent validity is evaluated by assessing the level of association between scores on the adapted scale and those on a comparator scale that measures related constructs. For the present investigation the NHP is used as the comparator instrument as it has been widely used in studies of growth hormone deficiency [27-29]. The NHP consists of six sections; energy level, pain, emotional reactions, sleep, social isolation and physical mobility. A unidimensional index of impairment - the NHPD - can also be derived from a subset of NHP items [30]. High scores on the NHP sections indicate worse health status. Unfortunately, no validated version of the NHP is available for Slovakia. Spearman rank correlation coefficients are employed to test the level of association between QoL-AGHDA scores and those on the NHP sections.

Known-group validity is assessed by testing the ability of a measure to distinguish between groups of people that differ according to a factor that is known, or suspected, to influence scores. Different information was routinely collected in each of the countries included in the study. In the Czech Republic patients' self-perceived general health information was available, rated: Excellent, very good, good, fair or poor. For the Polish and Serbian populations responses from two visual analogue scales (VASs) included in the KIMS database were available. These assessed physical activity during leisure time and satisfaction with physical activity during leisure time. Respondents were divided into two groups for each of these VASs - those scoring above the median and those scoring below the median. In the Czech, Polish and Serbian adaptations item 1 of the NHP: 'I am tired all the time' (yes/no) was also used to distinguish between participants given the importance of fatigue in GHD. No known-group data were available for the Slovakian adaptation.

For the known-group analyses, Mann-Whitney U Tests were employed for comparisons between two groups. Where more than two groups were compared Kruskal-Wallis one-way analysis of variance tests were applied.

## Results

### Translations

Demographic details for the bilingual and lay panels are shown in Table 1.The bilingual panels in each country were able to translate all instructions, response options and items without any serious difficulties. Where difficulties finding the correct wording for items were found alternative phrases were sent for consideration by the lay panels.

**Table 1:** Demographics for the translations, cognitive debriefing interviews and postal validation survey

	Czech Republic	Poland	Serbia	Slovakia
**Bilingual Translation Panel**				
n	6	6	5	6
**Gender**				
Male (%)	2 (33.3)	2 (33.3)	2 (40.0)	1 (16.7)
Female (%)	4 (66.7)	4 (66.7)	3 (60.0)	5 (83.3)
**Age (years)**				
Mean (SD)	32.6	35.3 (7.1)	42.6 (12.5)	31.3 (7.4)

**Lay Translation Panel**				
n	6	6	5	6
**Gender**				
Male (%)	5 (83.3)	3 (50.0)	2 (40.0)	3 (50.0)
Female (%)	1 (16.7)	3 (50.0)	3 (60.0)	3 (50.0)
**Age (years)**				
Mean (SD)	32.6 (13.0)	45.8 (10.4)	40.0 (14.5)	45.2 (18.8)

**Cognitive Debriefing Interviews**				
n	15	15	15	18
**Gender**				
Male (%)	10 (66.0)	6 (40.0)	7 (46.7)	9 (50.0)
Female (%)	5 (33.0)	9 (60.0)	8 (53.3)	9 (50.0)
**Age (years)**				
Mean (SD)	40.0 (15.9)	37.3 (16.4)	53.9 (12.2)	45.8 (17.8)

**Postal Validation Survey**				
n	100	85	34	106
**Gender**				
Male (%)	44 (62.9)	44 (51.8)	21 (61.8)	65 (61.3)
Female (%)	26 (37.1)	41 (48.2)	13 (38.2)	41 (38.7)
**Age (years)**				
Mean (SD)	42.6 (13.3)	49.4 (17.2)	43.7 (14.0)	40.1 (13.8)

All lay panels were able to choose between alternative translations sent by the bilingual panel. Some additional changes to item wording were also suggested by the lay panels to increase clarity or to make the phrases more colloquial. Care was taken to ensure that the suggested changes of wording did not alter the meaning of the original English items.

### Cognitive debriefing interviews

Interviewee details are provided in Table 1. The cognitive debriefing interviews were to assess the face and content validity of the measures. The samples included individuals with GH deficiency representative of the target population and had a good range of ages and gender. Participants generally reported finding the QoL-AGDHA to be a simple questionnaire that was easy to complete, with straightforward response options. Items were predominantly reported to be clear and relevant and none of the items were found to be badly worded or difficult to understand. Importantly, none of the patients stated that any important aspects of their experience had been omitted. Individual patients reported difficulties with specific items but these were found to be idiosyncratic rather than commonly expressed views.

### Postal validation survey results

Details of the samples included in the postal validation surveys are shown in Table 1. Information on gender was missing for 30 patients in the Czech Republic due to an error in the administration of the postal surveys in that country. Good sample sizes were obtained in the Czech Republic, Poland and Slovakia. Fewer patients were recruited in Serbia.

#### Questionnaire descriptive scores

QoL-AGHDA and NHP scores are shown in Table 2. Mean scores were in the low to mid range of the scales with a minor floor effect in some of the adaptations (Table 3).

**Table 2:** Questionnaire descriptive scores

Mean (SD)	Czech Republic	Poland	Serbia	Slovakia
**QoL-AGHDA (Time 1)**	7.0 (6.2)	9.2 (7.3)	6.2 (5.9)	10.1 (6.4)
**NHP (Time 1)**				
Energy level	27.5 (35.5)	40.5 (39.1)	18.6 (34.0)	-
Pain	5.3 (13.1)	16.2 (20.0)	12.9 (27.6)	-
Emotional reactions	18.2 (24.0)	30.9 (28.9)	17.5 (23.9)	-
Sleep	17.7 (25.4)	32.4 (33.9)	19.4 (29.3)	-
Social isolation	14.1 (25.8)	21.8 (30.6)	7.1 (16.2)	-
Physical mobility	7.0 (12.2)	19.9 (21.4)	15.4 (24.0)	-
NHPD	3.6 (4.0)	6.4 (5.2)	3.4 (4.6)	-

**Table 3:** Floor effects (% scoring minimum) for the QoL-AGHDA & NHP

	Czech Republic	Poland	Serbia	Slovakia
**QoL-AGHDA (Time 1)**	13.0	9.4	17.6	4.7
**NHP (Time 1)**				
Energy level	48.0	36.5	70.6	-
Pain	68.0	45.9	73.5	-
Emotional reactions	37.0	25.9	47.1	-
Sleep	48.0	35.3	61.8	-
Social isolation	58.0	55.3	76.5	-
Physical mobility	56.0	30.6	52.9	-
NHPD	21.0	18.9	35.3	-

#### Internal consistency

Cronbach's alpha coefficients are also shown in Table 3. The coefficients were high in all language adaptations indicating adequate inter-relatedness of items. All CITC coefficients were between 0.2 - 0.8 in the Serbian, Polish and Slovakian adaptations. Two items in the Czech Republic version had a CITC outside the ideal range. The overall internal consistency was still high for this version. The inclusion of these two items did not significantly lower the internal consistency of the scale.

#### Test retest reliability

Test-retest reliability is shown in Table 4. The results showed that all adaptations had good reproducibility.

**Table 4:** Internal consistency and reproducibility of the QoL-AGHDA adaptations

	Czech Republic	Poland	Serbia	Slovakia
**Alpha coefficient **(n)	.91 (85)	.91 (70)	.91 (31)	.89 (92)
**Test-retest reliability**	.91 (70)	.91 (84)	.88 (34)	.93 (72)

#### Questionnaire scores associated with demographic factors

No significant differences in QoL-AGHDA scores were found associated with gender or age.

#### Convergent validity

Correlations between QoL-AGHDA and NHP section scores are shown in Table 5. The pattern of associations was similar across languages. The only relation that showed some deviation between languages was between QoL-AGHDA scores and those on the NHP Social isolation section which correlated more weakly in the Serbian adaptation. As expected, QoL-AGHDA scores were closely associated with the Energy level and Emotional reactions sections of the NHP reflecting the impact of these aspects of health status on patients with GHD. The QoL-AGHDA also correlated highly with the NHPD reflecting the distress associated with the illness. QoL-AGHDA scores correlated only moderately with the other NHP sections - particularly the more physical aspects of the condition.

**Table 5:** Correlation between QoL-AGHDA and NHP section scores

NHP section	Czech Republic (n = 85-86)	Poland (n = 73-80)	Serbia (n = 32-34)
**Energy level**	.69	.68	.71
**Pain**	.38	.40	.42
**Emotional reactions**	.74	.83	.71
**Sleep**	.46	.41	.45
**Social isolation**	.65	.57	.32
**Physical mobility**	.47	.46	.53
**NHPD**	.87	.81	.73

All correlations were significant at the 0.01 level (2-tailed).

#### Known-group validity

**Figure 1:**
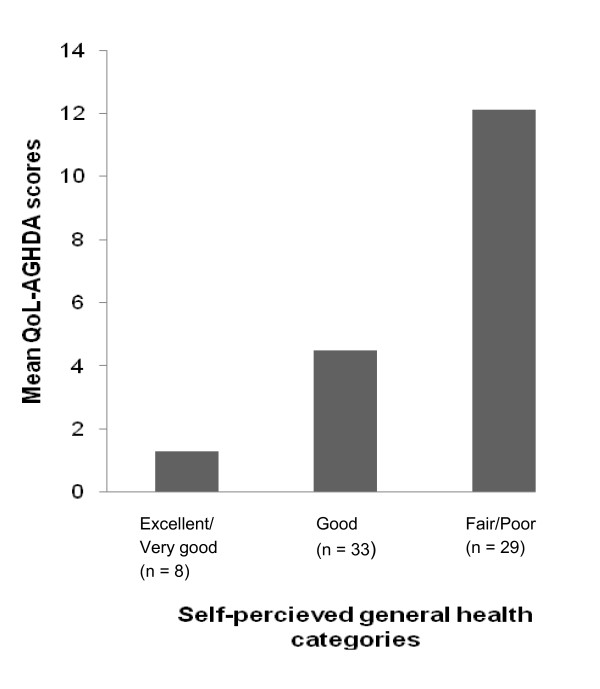
**Mean QoL-AGHDA scores by self-perceived general health in the Czech Republic (p < .01)**.

**Figure 2:**
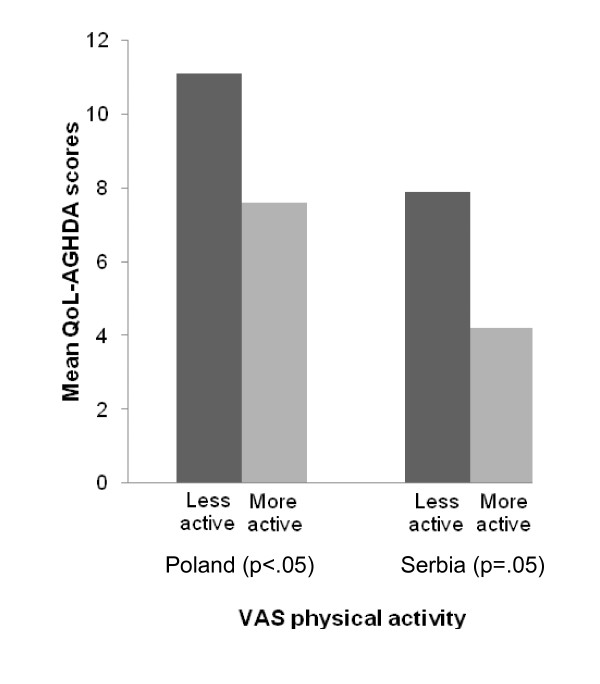
**Mean QoL-AGHDA scores by self-reported physical activity (Poland and Serbia)**.

**Figure 3:**
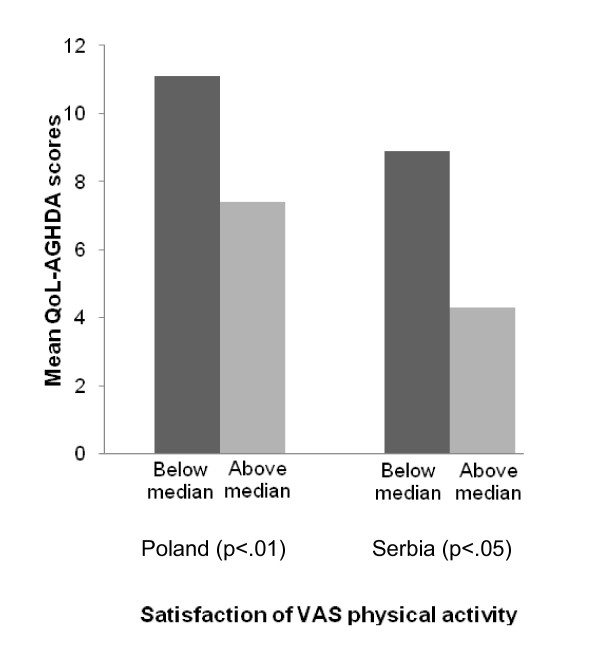
**Mean QoL-AGHDA scores by selef-perceived satisfaction of VAS physical activity (Poland and Serbia)**. VAS scores below the median indicate less satisfaction with physical activity

**Figure 4:**
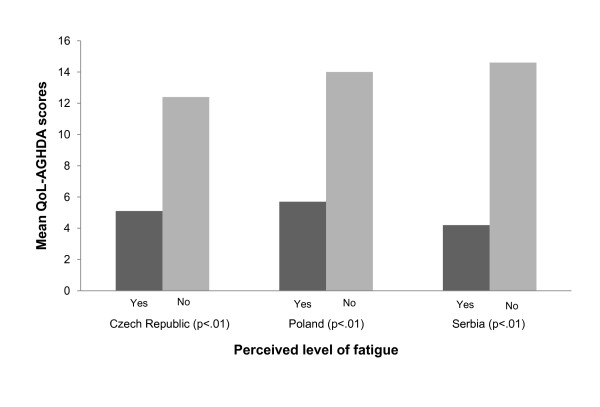
**Mean QoL-AGHDA scores by perceived fatigue**. Perceived fatigue is based on item 1 of the NHP 'I am tired all the time'.

Results of the known-group validity assessments are shown in Figures 1 to 4. In the Czech Republic the QoL-AGHDA was able to distinguish between participants based on their perceived general health (Figure 1). Individuals with worse perceived health had higher QoL-AGHDA scores. The Polish and Serbian adaptations were able to distinguish between participants based on their level of activity as measured by the self-report VAS (Figure 2) and by participants' satisfaction with their level of activity (Figure 3). Individuals with lower activity levels and those who were less satisfied had higher QoL-AGHDA scores. The Czech, Polish and Serbian adaptations were all able to distinguish between participants based on the NHP item 'I feel tired all the time' (Figure 4). Individuals who confirmed this item had higher QoL-AGHDA scores, indicating poorer QoL.

## Discussion

This paper describes the development of four new Slavic versions of the QoL-AGHDA. All four were shown to be internally consistent and to have good reproducibility. Clear evidence of the validity of the versions for the Czech Republic, Poland and Serbia is also presented although more evidence is necessary of the validity of the Slovakian adaptation. The QoL-AGHDA is now available in 14 languages thereby increasing its value as an outcome measure. It is widely used in clinical studies, in an international research database and for determining whether patients in the UK receive replacement therapy. It is to be hoped that evidence collected with the QoL-AGHDA in these new countries will have an influence on the availability of GH replacement therapy for local patients.

Few difficulties were found in translating the QoL-AGHDA into these new languages. This finding supports previous adaptation studies that have shown that there were few problems in adapting UK measures into Slavic languages using the dual panel methodology [23]. The present adaptation methodology is particularly effective in overcoming language differences as the questionnaire goes through several stages of refinement during the adaptation process. Quality is built into the process at each stage rather than depending on simple back translations [21]. The measure is first translated by a group of bilingual individuals and then tested for acceptability with a group of lay people. As these two stages involve groups of people working together to reach a consensus the adaption has the benefit of involving several individuals rather than one or two professional translators. This process also ensures that the wording of the questionnaire is less 'professional' and better targeted to patients [22]. The cognitive debriefing interview stage then checks that the measure is acceptable and relevant to local people with GH deficiency.

Although the adaptation of the QoL-AGHDA appeared to work well in each language there may still be cultural differences that affect how important each item is to participants. These differences could then affect the scores obtained by patients in the different countries. Such issues are rarely recognized or addressed by researchers adapting patient reported outcome measures (PROs). Previous studies have clearly shown that there can be systematic differences in the likelihood of affirming items in different countries (even within Western cultures) [31-33]. Such cultural differences are difficult to overcome and it is, as yet, unknown to what degree these differences may skew the results of international studies where data from multiple countries are combined. It is potentially possible through the application of Rasch analysis to plot scores from different countries on the same underlying metric and then make adjustments to control for cultural differences [32].

Although it is possible to adjust scores using Rasch analysis it is unclear how effective this would be when combining data from cultures that are very different. Issues that are important to patients in Europe may be of relatively little concern to those in Asia or Africa and vice versa. This means that even a good conceptual adaptation of a measure may contain many issues that are not important and may miss issues that are crucial in a different culture. Where the intention is to combine data from very different cultures it may well be necessary to use different versions of the same measure based on the same measurement model. Co-calibration of scores on the different measures using Rasch analysis would make it possible to combine data collected with the different versions of the measure.

Scores on the QoL-AGHDA were shown to be free from bias associated with age or gender. Moderately high levels of floor effects (respondents scoring 0) were found in the Czech and Serbian adaptations. It is likely that this reflects the mild nature of the samples obtained in these countries. This is supported by the high levels of floor effects observed for the NHP scales. Internal consistency and reproducibility of the new language versions were good indicating that the scales produce little measurement error. Scores on the scales also correlated as expected with NHP scores with the highest associations being with the Energy level and Emotional reaction sections - issues that are known to be important to patients with GHD. The relation between the NHP Social isolation and QoL-AGHDA scores was weaker in the Serbian adaptation than for the Czech and Polish adaptations. However, this section of the NHP is known to have cross-cultural problems [25]. As adult GH deficiency is a relatively rare condition and these countries (with the exception of Poland) have relatively small populations, recruiting samples for the validation surveys proved challenging. Consequently, further evidence is needed of the construct validity of these new adaptations of the QoL-AGHDA but the results to date are promising and similar to those found for other language adaptations of the measure [20]. It is hoped that researchers in these countries will employ the QoL-AGHDA in future studies to help establish further the scales' validity.

The study would also have benefitted from being able to employ the same comparator measures for each adaptation. However, the use of PROs in this region is relatively new and few measures have been adequately translated and/or adequately validated for these countries. Furthermore, the different centres involved in the studies collected different information about the samples.

## Conclusions

The adaptation of the QoL-AGHDA for use in the Czech Republic, Poland, Serbia and Slovakia was successful. All measures were easily adapted and showed excellent internal consistency and reproducibility. Good evidence of construct validity was found for the Czech, Polish and Serbian versions. Further validation of the Slovakian version would be helpful.. The new measures offer greater scope for the investigation of GHD in multinational clinical trials involving Eastern European countries. They will also prove valuable in monitoring the long-term efficacy and safety of growth hormone replacement therapy in these countries.

## Abbreviations

QoL: Quality of Life; PRO: patient reported outcome; GHD: Growth Hormone Deficiency; NHP: Nottingham Health Profile; The QoL AGHDA: The Quality of Life in Adult Growth Hormone Deficiency Assessment.

## Competing interests

The authors declare that they have no competing interests.

## Authors' contributions

SPM was involved with the design of the study, interpretation of the data and contributed to the manuscript. JW, JT and SRC were involved in the analysis and interpretation of the data and reviewed and contributed to the manuscript. VH, MK-L, VP and MP were involved with the acquisition of the data and reviewed and contributed to the manuscript. MK-H was involved with the design of the study, interpretation of the data and reviewed and contributed to the manuscript. All authors have read and approved the final manuscript.
